# Genetic analysis of the roles of *agaA*, *agaI*, and *agaS* genes in the N-acetyl-D-galactosamine and D-galactosamine catabolic pathways in *Escherichia coli* strains O157:H7 and C

**DOI:** 10.1186/1471-2180-13-94

**Published:** 2013-05-01

**Authors:** Zonglin Hu, Isha R Patel, Amit Mukherjee

**Affiliations:** 1 Division of Molecular Biology, Office of Applied Research and Safety Assessment, Center for Food Safety and Applied Nutrition, U.S. Food and Drug Administration, Laurel, MD 20708, USA

## Abstract

**Background:**

The catabolic pathways of N-acetyl-D-galactosamine (Aga) and D-galactosamine (Gam) in *E. coli* were proposed from bioinformatic analysis of the *aga/gam* regulon in *E. coli* K-12 and later from studies using *E. coli* C. Of the thirteen genes in this cluster, the roles of *agaA*, *agaI,* and *agaS* predicted to code for Aga-6-P-deacetylase, Gam-6-P deaminase/isomerase, and ketose-aldolase isomerase, respectively, have not been experimentally tested. Here we study their roles in Aga and Gam utilization in *E. coli* O157:H7 and in *E. coli* C.

**Results:**

Knockout mutants in *agaA*, *agaI,* and *agaS* were constructed to test their roles in Aga and Gam utilization. Knockout mutants in the N-acetylglucosamine (GlcNAc) pathway genes *nagA* and *nagB* coding for GlcNAc-6-P deacetylase and glucosamine-6-P deaminase/isomerase, respectively, and double knockout mutants Δ*agaA* Δ*nagA* and ∆*agaI* ∆*nagB* were also constructed to investigate if there is any interplay of these enzymes between the Aga/Gam and the GlcNAc pathways. It is shown that Aga utilization was unaffected in Δ*agaA* mutants but Δ*agaA* Δ*nagA* mutants were blocked in Aga and GlcNAc utilization. *E. coli* C Δ*nagA* could not grow on GlcNAc but could grow when the *aga/gam* regulon was constitutively expressed. Complementation of Δ*agaA* Δ*nagA* mutants with either *agaA* or *nagA* resulted in growth on both Aga and GlcNAc. It was also found that Δ*agaI*, Δ*nagB*, and ∆*agaI* Δ*nagB* mutants were unaffected in utilization of Aga and Gam. Importantly, Δ*agaS* mutants were blocked in Aga and Gam utilization. Expression analysis of relevant genes in these strains with different genetic backgrounds by real time RT-PCR supported these observations.

**Conclusions:**

Aga utilization was not affected in Δ*agaA* mutants because *nagA* was expressed and substituted for *agaA*. Complementation of Δ*agaA* Δ*nagA* mutants with either *agaA* or *nagA* also showed that both *agaA* and *nagA* can substitute for each other. The ∆*agaI*, ∆*nagB*, and ∆*agaI* ∆*nagB* mutants were not affected in Aga and Gam utilization indicating that neither *agaI* nor *nagB* is involved in the deamination and isomerization of Gam-6-P. We propose that *agaS* codes for Gam-6-P deaminase/isomerase in the Aga/Gam pathway.

## Background

**Figure 1:**
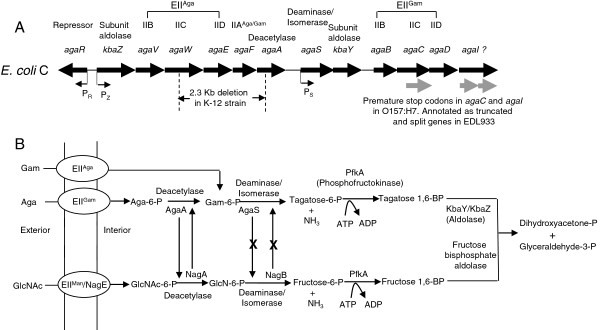
**The *aga/gam *regulon and the Aga, Gam, and GlcNAc pathways in *E. coli*. **(**A**) The genetic map (not drawn according to scale) shows the 13 genes and the protein products that they code for in the 12.3 Kb *aga/gam *cluster in *E. coli *C. *The agaI *gene was predicted to code for Gam-6-P deaminase/isomerase but this study and that of Leyn et al. [24] shows that *agaS *code for this deaminase. The question mark next to *agaI *indicates that the function of this gene is now uncertain. P_R., _P_Z,_ and P_S _are the promoters and the arrows indicate the direction of transcription. The 2.3 Kb deletion in the K-12 strain is shown and the truncated *agaC* gene and the split *agaI *gene as annotated in strain EDL933 are shown in gray arrows. (**B**) The Aga/Gam and the GlcNAc pathways are depicted in this figure. The only change from what was known before about the Aga/Gam pathway [1,6] is that AgaS carries out the deamination step and not AgaI as was known before. The GlcNAc pathway is shown to indicate the interplay between AgaA and NagA but not between AgaS and NagB as shown from this study. The upward vertical arrow from NagA indicates that it can substitute for AgaA and the downward vertical arrow indicates that AgaA can substitute for NagA when it is over-expressed. The upward vertical arrow from NagB with an X in the middle and a similar downward arrow from AgaS indicate that AgaS and NagB do not substitute for each other.

The pathway for utilization of the amino sugar, N-acetyl-D-galactosamine (Aga), in *Escherichia coli* was proposed from bioinformatic analysis of the genome sequence of *E. coli* K-12 [1] and by drawing parallels to the catabolic pathway of the related amino sugar, N-acetyl-D-glucosamine (GlcNAc) [2-5]. A more complete understanding of the Aga pathway came upon studying it in *E. coli* C because it has the whole set of 13 genes for the utilization of both Aga and D-galactosamine (Gam) and is therefore Aga^+^ Gam^+^ (Figure 1) [6]. The K-12 strain, on the other hand, is Aga^-^ Gam^-^ because it has a 2.3 Kb deletion leading to the loss and truncation of genes that are needed for Aga and Gam utilization [6]. The *aga/gam* regulon and the Aga/Gam pathway in *E. coli* has been described before [1,6] and is shown in Figure 1. The transport of Aga and Gam into the cell as Aga-6-P and Gam-6-P, respectively, is mediated by their respective Enzyme II (EII) complexes of the phosphoenolpyruvate: carbohydrate phosphotransferase system (PTS) [7,8] and is further catabolized as shown in Figure 1B. The *agaI* gene was predicted to code for Gam-6-P deaminase/isomerase that converts Gam-6-P to tagatose-6-P and NH_3_[1,6] but as shown here later this is not so. The proposed Aga/Gam pathway is analogous to the better studied GlcNAc pathway (Figure 1B) [2-5]. GlcNAc, a PTS sugar, is transported by the GlcNAc PTS coded by *nagE* or by the mannose PTS coded by *manXYZ.* The resulting GlcNAc-6-P is deacetylated by GlcNAc-6-P deacetylase coded by *nagA* to glucosamine-6-P (GlcN-6-P). GlcN-6-P is then deaminated and isomerized by *nagB* encoded GlcN-6-P deaminase/isomerase forming fructose-6-P and NH_3_.

Although the functions of the genes in the *aga/gam* cluster were initially surmised from *in silico* studies, there are some experimental data now that support the predicted functions of ten of the thirteen genes. Genetic and transport studies in *E. coli* C and in *E. coli* K-12 support the prediction of the PTS genes in the *aga/gam* cluster [6,9]. The induction of tagatose 1, 6-BP aldolase activity by Aga and Gam along with other complementation studies demonstrates that *kbaY* codes for the aldolase [6,10] and *kbaZ* codes for the subunit that is needed for full activity and stability *in vitro*[10]. It has been shown that the *agaR* encoded repressor binds to promoters upstream of *agaR, kbaZ,* and *agaS* (Figure 1) [11]. That *agaA* codes for Aga-6-P deacetylase was indirectly implied because Aga utilization was unaffected in a *nagA* mutant [6]. The assigned role of the *agaI* gene as Gam-6-P deaminase/isomerase had not been tested and what, if any, role the *agaS* gene plays in the Aga/Gam pathway was not known although it was predicted to code for a ketose-aldose isomerase [1,6,11].

The interest in the Aga/Gam pathway stems from our earlier finding that isolates of the foodborne pathogen, *E. coli* O157:H7, from a spinach outbreak could not utilize Aga because of a point mutation in EIIA^Aga/Gam^ (Gly91Ser) [12]. We also pointed out that *E. coli* O157:H7, strains EDL933 and Sakai, harbor two additional point mutations in *agaC* and *agaI.* Both mutations change a CAG codon coding for glutamine to TAG, an amber stop codon: one in the eighth codon of the *agaC* gene that codes for EIIC^Gam^; and the other in the 72nd codon of the *agaI* gene that had been proposed to code for Gam-6-P deaminase/isomerase. Although these two mutations reside in both EDL933 and Sakai strains, the annotations are different in these two strains. In EDL933, *agaC* is annotated as a 5’ truncated gene and *agaI* is annotated as a split gene (Figure 1) whereas, in the Sakai strain they are not annotated at all [12]. In *E. coli* O157:H7, the amber mutation in *agaC* affects EIIC^Gam^ which explains the Gam^-^ phenotype but the mutation in *agaI* does not affect utilization of Aga as the sole source for carbon and nitrogen [12]. The obvious question that arises is how does this happen without an active Gam-6-P deaminase/isomerase. *E. coli* K-12 is Aga^-^ Gam^-^ but isolation of suppressor mutants of *E. coli* K-12 with mutations in the GlcNAc regulon that were Aga^+^ Gam^-^ has been reported [6]. These mutants transported Aga by the GlcNAc PTS and since *nagA* was required for Aga utilization it was inferred that NagA deacetylated Aga-6-P. Based on these findings we had hypothesized, by analogy, that *nagB* might similarly substitute for *agaI* in *E. coli* O157:H7 enabling it to utilize Aga as carbon and nitrogen source [12] and here we test this hypothesis.

In this study a genetic approach was taken to delineate the roles of *agaA*, *agaI*, and *agaS* in the Aga/Gam pathway in *E. coli*. These studies were carried out in parallel using *E. coli* O157:H7 strain EDL933 and in *E. coli* C. *E.* coli C was chosen because, unlike *E. coli* O157:H7, it does not have the mutations in *agaC* and *agaI* and also because it is Gam^+^, one can study the roles of *agaI* and *agaS* in Gam utilization. We show using knockout mutants and by complementation studies that *agaA* is not essential for Aga utilization and that AgaA and NagA can function as deacetylases in both the Aga and the GlcNAc pathways. The phenotype of deleting *agaR* in a *nagA* strain was also studied but only in *E. coli* C. Expression analysis of the relevant genes of these two pathways by quantitative real time RT-PCR (qRT-PCR) validated our conclusions. We also show that in the absence of *agaI*, *nagB* or both *agaI* and *nagB*, utilization of Aga and Gam is not affected which contradicts our initial hypothesis that *nagB* might substitute for the absence of agaI in *E. coli* O157:H7 [12]. Finally, we show that utilization of both Aga and Gam is blocked in *agaS* knockout mutants and we propose that this gene codes for Gam-6-P deaminase/isomerase. [Part of this work was presented by the authors as a poster in the 112th General Meeting of ASM, San Francisco, June 16th-19th, 2012: A Genetic Approach to Study Utilization of N-Acetyl-D-Galactosamine and D-Galactosamine in *Escherichia coli* Strains O157:H7 and C (Abstract K-1351)].

## Results and Discussion

### Growth of Δ*agaA*, Δ*nagA*, and Δ*agaA* Δ*nagA* mutants on Aga and GlcNAc

**Figure 2:**
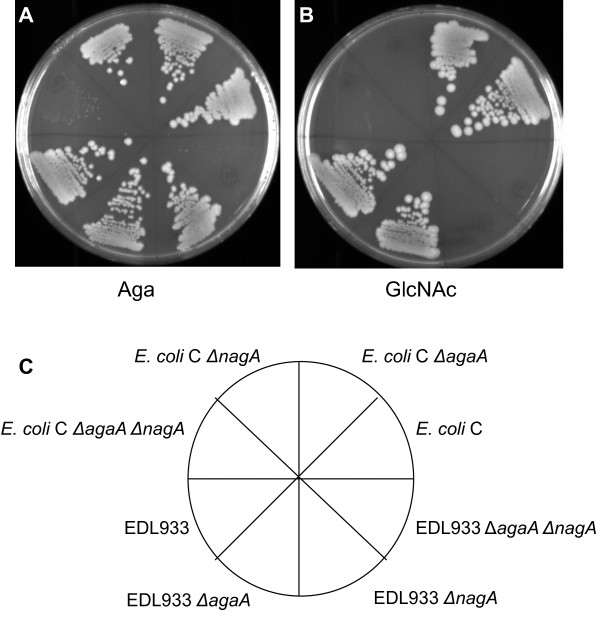
**Growth of EDL933**, ***E. coli *C, and their mutants on Aga and GlcNAc. **EDL933, *E. coli *C, and the indicated knockout mutants derived from them were streaked out on MOPS minimal agar plates containing Aga (**A**) and GlcNAc (**B**) and incubated at 37°C for 48 h. The description of the strains in the eight sectors of the plates is indicated in the diagram below (**C**).

The role of the *agaA* gene in Aga utilization was tested by constructing *agaA* deletion mutants in EDL933 and in *E. coli* C and analyzing them for growth on Aga and GlcNAc minimal medium plates. Unexpectedly, the utilization of Aga was unaffected in both Δ*agaA* mutant strains (Figure 2A). However, the Δ*agaA* mutants were unaffected in GlcNAc utilization (Figure 2B) and this was not unexpected because the *nagA* gene is intact. As mentioned above, earlier genetic studies implied that Aga can be utilized by the GlcNAc pathway provided *nagA* is present [6]. Assuming that an unknown deacetylase is not involved in Aga-6-P deacetylation, the most likely explanation how Δ*agaA* mutants grew on Aga would be that Aga-6-P is deacetylated by NagA. Therefore, the presence of either *agaA* or *nagA* should be sufficient for growth on Aga. To test this unequivocally, Δ*nagA* mutants and double knockout mutants, Δ*agaA* Δ*nagA*, of EDL933 and *E. coli* C were constructed and examined for Aga and GlcNAc utilization. EDL933 Δ*nagA* and *E. coli* C Δ*nagA* grew on Aga but did not grow on GlcNAc (Figures 2A and 2B). These results essentially confirmed earlier reports that *nagA* mutants of *E.* coli K-12 cannot grow on GlcNAc but can grow on Aga [2,4,6]. Phenotypic microarray experiments using the Biolog system [13] also showed that the Δ*nagA* mutants could not utilize N-acetylmannosamine (ManNAc) and N-acetylneuraminic acid (data not shown) because all three utilization pathways converge to the common intermediate GlcNAc-6-P [5]. When the Δ*agaA* Δ*nagA* double knockout mutant strains of EDL933 and *E. coli* C were examined for growth on GlcNAc and Aga it was found that both strains did not grow on GlcNAc as expected but importantly, these mutants also did not grow on Aga (Figures 2A and 2B). These results indicate that *agaA* is not essential for Aga utilization because *nagA* can substitute for *agaA* and therefore the presence of either *agaA* or *nagA* is sufficient for Aga utilization.

### Quantitative real time RT-PCR analysis reveal that *nagA* and *nagB* are expressed in Δ*agaA* mutants grown on Aga

To investigate if NagA is induced in Δ*agaA* mutants when grown on Aga we examined the relative expression levels of *agaA* and *nagA* in wild type, Δ*agaA*, and Δ*nagA* strains of EDL933 and *E. coli* C grown on different carbon sources by qRT-PCR. The expression of the *agaS* gene was also examined as a second gene of the *aga/gam* regulon that is under the control of the second promoter, P_s*,*_ and similarly *nagB* was chosen as a second gene of the *nag* regulon. Relative expression levels of genes in wild type and mutant strains of EDL933 and *E. coli* C grown on Aga and GlcNAc were calculated with respect to that of the expression of the corresponding genes in wild type strains grown on glycerol. As shown in Table 1, growth on Aga induced *agaA* and *agaS* about 375 and 500-fold, respectively, in EDL933 and about 30 and 60-fold, respectively, in *E. coli* C. The *nagA* and *nagB* genes were not induced by Aga in either strain. Growth on GlcNAc induced *nagA* and *nagB* about 12 and 24-fold, respectively, in EDL933 and 16 and 23 fold, respectively, in *E. coli* C. In presence of GlcNAc, *agaA* and *agaS* were not induced in EDL933, but in *E. coli* C the induction was minimal, which is less than 10% of that in Aga grown cells. In Aga grown cells the induction of *agaA* and *agaS* was about 12 and 8-fold higher, respectively, in EDL933 than in *E. coli* C but the levels of induction of *nagA* and *nagB* in both strains grown on GlcNAc were comparable (Table 1). Earlier studies using single copy lysogenic derivatives of *E. coli* K-12 harboring P_z-_*lacZ* and P_s_-*lacZ* transcriptional fusions showed that the P_z_ and the P_s_ promoters were induced 5 and 20-fold, respectively, upon growth on Aga in minimal medium containing 0.2% casamino acids but growth in GlcNAc did not induce expression from these promoters [11]. These levels of induction from the P_z_ and P_s_ promoters are much lower than what is reported here (Table 1) and that is most likely because of different experimental conditions, strains used, and assay method employed but the essential pattern of induction is the same in both studies in that Aga induces the *aga/gam* regulon and GlcNAc does not. Growth of *E. coli* K-12 on GlcNAc results in the induction of the *nag* regulon that includes *nagBACD* in one operon and the divergently transcribed operon with the *nagE* gene coding for the GlcNAc transport protein, EII^Nag^[3]. However, it has also been reported that in *E. coli* K92 the GlcNAc transport protein is induced by both GlcNAc and Aga [9]. Although, in our qRT-PCR assays we only examined *nagA* and *nagB* expression and not *nagE* expression, the expression pattern of *nagA* and *nagB* should reflect that of *nagE* expression because they are all part of the *nag* regulon [3]. Therefore, unlike what was observed in *E.* coli K92 [9], our data (Table 1) show that in EDL933 and *E. coli* C *nagA* and *nagB* were induced only by GlcNAc and not by Aga and thereby it would be reasonable to conclude that *nagE* was also not induced by growth on Aga. This discrepancy between our observation with two strains of *E. coli*, EDL933 and C, and that observed in *E. coli* strain K92 [9] is probably due to strain difference.

**Table 1:** **Analysis of gene expression in EDL933, *E. coli *C, and their mutants by qRT-PCR**

Carbon Source^a^	Strain	Relative expression of genes in EDL933 and *E. coli C*^b^			
		*agaA*	*agaS*	*nagA*	*nagB*
Glycerol	EDL933/*E. coli C*	1/1	1/1	1/1	1/1
Aga	EDL933/*E. coli C*	375/32	495/62	1/1	1/1
GlcNAc	EDL933/*E. coli C*	1/3	1/3	12/16	24/23
Glycerol	EDL933 *∆agaA /E. coli *C *∆agaA*	ND/ND^c^	1/1	1/1	1/1
Aga	EDL933 *∆agaA /E. coli *C *∆agaA*	ND/ND	699/86	16/7	28/9
GlcNAc	EDL933 *∆agaA /E. coli *C *∆agaA*	ND/ND	5/3	12/9	20/13
Glycerol	EDL933*∆nagA */*E. coli *C *∆nagA*	2/0.5	2/0.2	ND/ND	61/19
Aga	EDL933*∆nagA */*E. coli *C *∆nagA*	820/179	917/93	ND/ND	8/2

^a ^Carbon source used for growth.

^b ^The relative expression values after the forward slash is that of *E. coli *C.

^c ^ND indicates not detected.

In ∆*agaA* mutants of EDL933 and *E. coli* C, the expression of *agaA* could not be detected, as expected, irrespective of the carbon source used for growth (Table 1). When these two ∆*agaA* mutants were grown on glycerol, the expression levels of *agaS*, *nagA*, and *nagB* were unchanged compared to that of the wild type strains grown on glycerol. When the ∆*agaA* mutants of EDL933 and *E.* coli C were grown on Aga, the induction of *agaS* was about 700-fold and 90-fold, respectively, which is140% higher than that in their parent strains grown on Aga (Table 1). Thus, the relative expression level of *agaS* was higher in ∆*agaA* mutants grown on Aga. In Aga grown ∆*agaA* mutants, *nagA* and *nagB* were significantly induced whereas, these genes were not induced at all in wild type strains grown on Aga. In fact, in Aga grown EDL933 ∆*agaA*, the relative expression levels of *nagA* and *nagB* were about 130% compared to that of their expressions in wild type EDL933 and EDL933 ∆*agaA* grown on GlcNAc. The relative expression levels of *nagA* and *nagB* in *E. coli* C ∆*agaA* grown on Aga were about 70% of that when grown on GlcNAc and 40% of that in *E. coli* C grown on GlcNAc. In GlcNAc grown EDL933 ∆*agaA*, the expression levels of *nagA* and *nagB* were about the same as that of EDL933 grown on GlcNAc and the expression of *agaS* is slightly elevated but it is only about 1% of that in Aga grown EDL933*.* In *E. coli* C ∆*agaA* grown on GlcNAc the expression levels of *nagA* and *nagB* were 40% of that in *E. coli* C and the expression of *agaS* is about 3-fold higher than that grown in glycerol but it is about 5% of the level expressed in Aga grown *E. coli* C and *E. coli* C ∆*agaA*. What is noteworthy is that unlike in Aga grown wild type EDL933 and *E. coli* C where *nagA* and *nagB* were not induced, their respective ∆*agaA* mutants when grown on Aga induced *nagA* and *nagB* to levels that were comparable to the induced levels in GlcNAc grown in the wild type and the ∆*agaA* mutants of these strains. Importantly, this data shows that NagA is indeed present in Aga grown Δ*agaA* mutants and therefore it lends additional support to the genetic data (Figure 2) from which we concluded that ∆*agaA* mutants of EDL933 and *E. coli* C were able to grow on Aga (Figure 2) because NagA can substitute for the absence of AgaA. This observation leads to the question how do Δ*agaA* mutants grown on Aga induce *nagA* and *nagB* and thereby the *nag* regulon. A probable explanation is that when Δ*agaA* mutants grow on Aga they accumulate Aga-6-P which induces the *nag* regulon and upon synthesis of NagA it deacetylates Aga-6-P. It has been shown that the inducer of the *nag* regulon is GlcNAc-6-P and not GlcN, GlcNAc, GlcN-6-P, and G-1-P [4]. There is also indirect evidence that Aga-6-P is the inducer of the *aga/gam* regulon [11] but whether Aga-6-P can also induce the *nag* regulon has not been demonstrated.

When *nagA* and *nagB* expression levels were examined in glycerol grown Δ*nagA* mutants it was found that expression of *nagA* was not detected as expected, and *agaA* and *agaS* were expressed at very low levels. However, *nagB* was induced 61-fold in EDL933 Δ*nagA* and 19-fold in *E. coli* C Δ*nagA* whereas, in their respective wild type parents grown on glycerol it was not induced (Table 1). These expression levels of *nagB* in glycerol grown EDL933 Δ*nagA* and *E. coli* C ∆*agaA* were about 250% and 80%, respectively, of their respective wild type strains grown in GlcNAc. This is significantly high considering that the expression of *nagB* remains at the uninduced levels in the wild type strains grown on glycerol. This phenomenon of *nagB* induction in *nagA* mutants of *E. coli* K-12 grown on glucose has been reported earlier [2,4]. It has been explained that this happens because of the endogenous synthesis of GlcNAc-6-P, the inducer of the *nag* regulon, that accumulates in *nagA* mutants which in turn induces the *nag* regulon [2,4]. It was also reported that this accumulated substance in Δ*nagA* mutants disappeared upon incubation of a cell extract with overexpressed GlcNAc-6-P deacetylase [4]. The Δ*nagA* mutants in this study were grown in glycerol and not in glucose as in the earlier reports [2,4] but that did not affect the endogenous synthesis of GlcNAc-6-P, as we still observed *nagB* induction in these mutants. What is interesting to note, however, is that when both the Δ*nagA* mutants were grown on Aga, the induced levels of *nagB* fell drastically to about 10% of that in glycerol grown Δ*nagA* mutants (Table 1). A very likely reason why this happens is that upon induction of *agaA* in Δ*nagA* mutants by Aga, the induced AgaA deacetylates the accumulated GlcNAc-6-P to GlcN-6-P thereby lowering the intracellular concentration of GlcNAc-6-P which results in turning down the expression of the *nag* regulon. This strongly suggests that AgaA can deacetylate GlcNAc-6-P in addition to Aga-6-P just like NagA can substitute for the absence of AgaA. Finally, in Aga grown EDL933 Δ*nagA* the induced levels of *agaA* and *agaS* were about 220% and 180%, respectively, of that in Aga grown EDL933 and likewise, in *E. coli* C Δ*nagA* grown on Aga, the induced levels of *agaA* and *agaS* were about 550% and 150%, respectively, of that in *E. coli* C grown on Aga. Why this happens remains to be investigated.

### Constitutive expression of the *aga/gam* regulon enables a Δ*nagA* mutant to grow on GlcNAc

**Figure 3:**
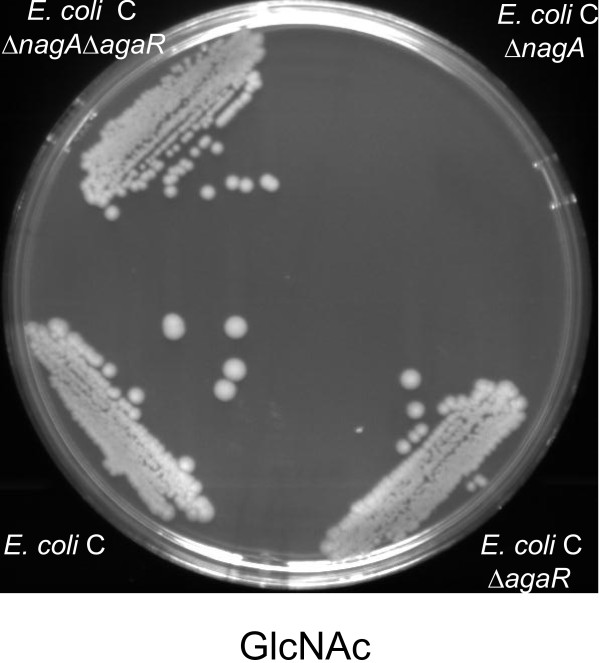
**Growth of *E. coli *C and mutants derived from it on GlcNAc. ***E. coli *C and the indicated mutants derived from it were streaked out on GlcNAc MOPS minimal agar plates and incubated at 37°C for 48 h.

The induction of *nagB* in Δ*nagA* mutants grown on glycerol and its repression when grown on Aga (Table 1) indicated that AgaA deacetylated GlcNAc-6-P. Unlike Δ*agaA* mutants which grew on Aga (Figure 2) because *nagA* was expressed in these mutants by Aga (Table 1), Δ*nagA* mutants did not grow on GlcNAc most likely because *agaA* is not expressed with GlcNAc (Figure 1). If this is true, then deleting the *agaR* gene, that codes for the repressor of the *aga/gam* regulon, in a Δ*nagA* mutant would result in the constitutive expression of the *aga/gam* regulon and thereby of *agaA* that would allow its growth on GlcNAc. Therefore, *agaR* deletion mutants in *E. coli* C and in *E. coli* C Δ*nagA* were constructed and examined for growth on GlcNAc. As shown in Figure 3, *E. coli* C and *E. coli* C Δ*agaR* grew on GlcNAc and the Δ*nagA* mutant did not grow but the double knockout strain, *E. coli* C Δ*nagA* Δ*agaR*, did indeed grow on GlcNAc. Phenotypic microarray [13] done with *E. coli* C Δ*nagA* Δ*agaR* also revealed that it regained the ability to utilize ManNAc and N-acetylneuraminic acid in addition to that of GlcNAc (data not shown) as their utilization is *nagA* dependent [5]. Analysis by qRT-PCR was done to confirm that *agaA* and *agaS* were constitutively expressed in *E. coli* C Δ*agaR* and in *E. coli* C Δ*nagA* Δ*agaR.* As shown in Table 2, *agaA* and *agaS* were expressed in *E. coli* C Δ*agaR* and *E. coli* C Δ*nagA* Δ*agaR* irrespective of the carbon source used for growth but *nagA* and *nagB* were induced only by GlcNAc and, as expected, *nagA* expression was not detected in *E. coli C* Δ*nagA* Δ*agaR*. In fact, *agaA* and *agaS* were induced higher in these Δ*agaR* mutants than that in Aga grown *E. coli* C, the only exception being that of *agaA* whose induction was slightly lower in GlcNAc grown *E. coli* Δ*agaR*. These results confirm that *agaA* and *agaS* and thereby the aga*/gam* regulon were constitutively expressed in *E. coli* C Δ*agaR* and in *E. coli* C Δ*nagA* Δ*agaR.* This demonstrates that constitutive synthesis of AgaA can substitute for NagA in a Δ*nagA* mutant and allow it to grow on GlcNAc (Figure 3) just as NagA can substitute for AgaA in a Δ*agaA* mutant (Figure 2 and Table 1). It is interesting to note that unlike in glycerol grown *E. coli* C Δ*nagA* where *nagB* was induced 19-fold (Table 1), in glycerol grown *E. coli* C Δ*nagA* Δ*agaR*, where *agaA* was constitutively expressed, the relative expression of *nagB* was down to 2-fold (Table 2) which is the same as that in Aga grown *E. coli* C Δ*nagA* (Table 1). Thus, either the induced expression of *agaA *in *E. coli* C Δ*nagA* by growth on Aga (Table 1) or the constitutive expression of *agaA* in glycerol grown *E. coli* C Δ*nagA* Δ*agaR* (Table 2), turns down *nagB* induction significantly*.* Both these experiments indicate that AgaA can deacetylate GlcNAc-6-P.

**Table 2:** **Analysis of gene expression in *E. coli *C, *∆agaR*, and *∆nagA ∆agaR *mutants by qRT-PCR**

Carbon Source^a^	Strain	Relative expression of genes in *E. coli C*				
		*agaA*	*agaS*	*nagA*	*nagB*	*agaR*
Glycerol	*E. coli C*	1	1	1	1	1
Aga	*E. coli C*	32	62	1	1	2
GlcNAc	*E. coli C*	3	3	16	23	2
Glycerol	*E. coli *C *∆agaR*	50	175	1	1	ND^b^
Aga	*E. coli *C *∆agaR*	57	177	1	1	ND
GlcNAc	*E. coli *C *∆agaR*	20	92	6	13	ND
Glycerol	*E. coli C ∆nagA∆agaR*	54	197	ND	2	ND
Aga	*E. coli C ∆nagA∆agaR*	74	224	ND	3	ND
GlcNAc	*E. coli C ∆nagA∆agaR*	47	148	ND	26	ND

^a ^Carbon source used for growth.

^b ^ND indicates not detected.

### Complementation studies reveal that *agaA* and *nagA* can function in both the Aga and the GlcNAc pathways

**Figure 4:**
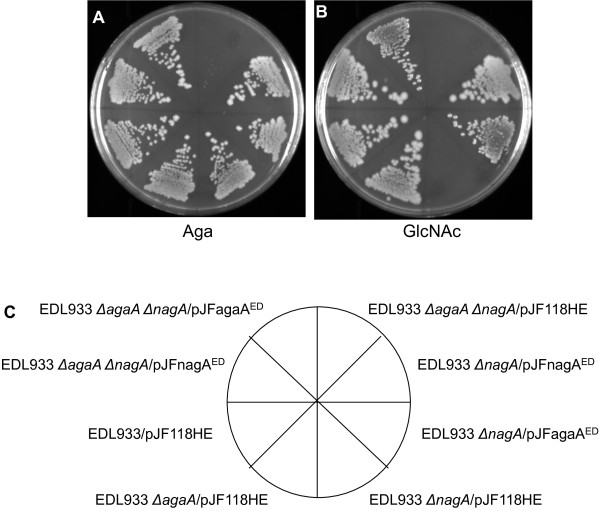
**Complementation of Δ*nagA *and Δ*agaA *Δ*nagA *mutants of EDL933 on Aga and GlcNAc plates. **Wild type EDL933 and knockout mutants derived from it harboring the indicated plasmids were streaked out on MOPS minimal agar plates with ampicillin containing Aga (**A**) and GlcNAc (**B**) and incubated at 37°C for 48 h. The description of the strains with various plasmids in the eight sectors of the plates is indicated in the diagram below (**C**).

The genetic and the qRT-PCR data described above show that *agaA* and *nagA* can substitute for each other. The relative expression levels in Table 1 show that in Aga grown Δ*agaA* mutants, *nagA* and *nagB* and thereby the *nag* regulon were induced and in *E. coli* C Δ*nagA* Δ*agaR*, *agaA* and *agaS* and thereby the whole *aga/gam* regulon were constitutively expressed. Although both regulons were turned on it is apparent that the expression of *nagA* in Δ*agaA* mutants and the expression of *agaA* in *E. coli* C *nagA* Δ*agaR* allowed growth on Aga and GlcNAc, respectively, and not the other genes of their respective regulons. In order to demonstrate that this is indeed so and to provide additional evidence that *agaA* and *nagA* can substitute for each other, we examined if both *agaA* and *nagA* would complement Δ*nagA* mutants to grow on GlcNAc and Δ*agaA* Δ*nagA* mutants to grow on Aga and GlcNAc. EDL933/pJF118HE and EDL933 Δ*agaA*/pJF118HE grew on Aga and GlcNAc, EDL933 Δ*nagA*/pJF118HE grew on Aga but not on GlcNAc, and EDL933 Δ*agaA* Δ*nagA*/pJF118HE did not grow on Aga and GlcNAc (Figures 4A and 4B). These results are the same as what was observed with these strains without pJF118HE (Figure 2). EDL933 Δ*nagA*/ pJFnagA^ED^ grew on GlcNAc which was expected but interestingly EDL933 Δ*nagA*/ pJFagaA^ED^ also grew on GlcNAc showing that *agaA* restored growth of a Δ*nagA* mutant on GlcNAc (Figure 4B). When EDL933 Δ*agaA* Δ*nagA* was complemented with either pJFnagA^ED^ or pJFagaA^ED^ growth was restored on both GlcNAc and xAga plates (Figures 4A and 4B). The plates shown in Figure 4 were incubated without IPTG indicating that the basal level of expression of NagA and AgaA from pJFnagA^ED^ and pJFagaA^ED^, repectively, were sufficient for complementation for growth on GlcNAc and Aga. Growth on GlcNAc and Aga plates at IPTG concentrations of 10, 50 and 100 μM was similar to that without IPTG indicating that higher levels of expression of *agaA* and *nagA* were not detrimental to the cells (data not shown). Identical results as those shown in Figure 4 were obtained in complementation experiments with *E. coli* C Δ*agaA*, Δ*nagA*, and Δ*agaA* Δ*nagA* mutants with plasmids, pJFagaA^C^ and pJFnagA^C^ (data not shown).

Thus far, several lines of evidence using knockout mutants, complementation studies with these mutants, and measuring the relative expression of relevant genes in these mutant strains and in the wild type strains indicate that NagA coded by *nagA* and AgaA coded by *agaA* can function in both the GlcNAc and Aga pathways. In this context it is pointed out that it was reported in *E. coli* K92, growth on Aga not only induced the Aga transport system but also induced the GlcNAc transport system [9]. From this observation it was proposed that an unidentified epimerase converts Aga-6-P to GlcNAc-6-P which then induces the GlcNAc transport system that is part of the *nag* regulon [9]. Our data differ in that, *nagA* and *nagB* and therefore the *nag* regulon were induced only in Δ*agaA* mutants and not in wild type *E. coli* C and EDL933 (Table 1). Furthermore, epimerases usually carry out substrate concentration dependent reversible reactions. Therefore, the high intracellular concentration of GlcNAc-6-P that accumulate in glucose grown *nagA* mutant (3.2 mM) [2], which should be about the same in our glycerol grown Δ*nagA* mutants (discussed above), should have epimerized to Aga-6-P. Aga-6-P which is the likely inducer of the *aga/gam* regulon [11] would then induce the *aga/gam* regulon but we show that it was not induced (Table 1). Instead, *nagB* was highly induced and *agaA* and *agaS* were induced only 2-fold in EDL933 Δ*nagA* but not in *E. coli* C Δ*nagA* (Table 1). Given the evidence that we have so far and that such an epimerase is yet to be identified, it seems unlikely that such an epimerase links the two pathways.

The *nagA* encoded GlcNAc-6-P deacetylase from *E. coli* K-12 has been purified and its enzymatic activity and properties are well established [14]. Therefore, the fact that *agaA* can substitute *nagA* in the utilization of GlcNAc shown by complementation studies (Figure 4) is strong evidence that *agaA* codes for a deacetylase. These observations indicate that both NagA and AgaA can act on substrates that are structurally closely related to their actual substrates. In a study by Plumbridge and Vimr [5] on the catabolic pathways of GlcNAc, ManNAc, and N-acetylneuraminic acid, where all of these amino sugars converge to GlcNAc-6-P and hence their utilization was *nagA* dependent it was argued that ManNAc-6-P is not deacetylated by NagA but instead isomerized to GlcNAc-6-P by the product of another gene, *yhcJ* . Their reasoning was that while both GlcNAc-6-P and ManNAc-6-P are N-acetyl substituted sugars at the C2 position, ManNAc-6-P is an epimer of GlcNAc-6-P at the C2 position and therefore makes it unlikely that NagA could position itself on the sugar molecule such that it has access to the acetyl group on both sides of the C2 atom. However, this argument would not hold true for Aga-6-P because it is an epimer of GlcNAc-6-P at the C4 position and so in both molecules the N-acetyl group is on the same side of the C2 position and therefore both NagA and AgaA could deacetylate Aga-6-P and GlcNAc-6-P as supported by the genetic complementation experiments (Figure 4).

### The utilization of Aga and Gam as carbon and nitrogen sources by *E. coli* is not affected by the absence of both *agaI* and *nagB*

While *E. coli* C and K-12 have an intact *agaI,* the *agaI* gene in *E. coli* O157:H7 has an amber mutation and yet it can utilize Aga. Four possible explanations can be proposed as to how *E. coli* O157:H7 can grow on Aga: i) *nagB* may substitute for the absence of *agaI*[12]; ii) the split ORFs in *agaI* are translated to form two polypeptide chains that form a functional enzyme; iii) the suppression of the amber codon by a suppressor tRNA leading to translation of a functional enzyme [15]; and iv) *agaI* and *nagB* are not essential for Aga and Gam utilization and the product of some other gene carries out this step in the pathway. These proposals were tested by constructing Δ*agaI,* Δ*nagB*, and Δ*agaI* Δ*nagB* mutants of EDL933 and *E. coli* C and examining their growth on Aga, Gam, and GlcNAc with and without NH_4_Cl. Growth of these mutants on plates with just the amino sugar without any added nitrogen source such as NH_4_Cl, would indicate that deamination of the Aga and Gam is taking place in the cell and hence there must be a functional deaminase/isomerase.

**Figure 5:**
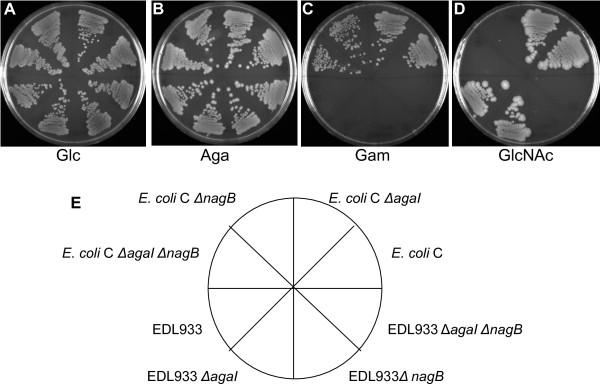
**Growth of EDL933, *E. coli *C, and mutants derived from them on different carbon sources. **EDL933, *E. coli *C, and the indicated knockout mutants derived from them were streaked out on MOPS minimal agar plates with glucose (**A**), Aga (**B**), Gam (**C**), and GlcNAc (**D**) with NH_4_Cl as added nitrogen source. All plates, except Gam containing plates, were incubated at 37°C for 48 h. Gam plates were incubated at 30°C for 72 to 96 h. The description of the strains in the eight sectors of the plates is indicated in the diagram below (**E**).

The wild type strains, EDL933 and *E. coli* C, and their Δ*agaI*, Δ*nagB*, and Δ*agaI* Δ*nagB* mutants were tested for growth on minimal medium plates containing glucose (Glc) as a control, Aga, Gam, and GlcNAc with and without NH_4_Cl as added nitrogen source. EDL933 and *E. coli* C and all the three mutants grew on Glc with NH_4_Cl (Figure 5A) but as expected none of them grew on Glc without a nitrogen source (data not shown). EDL933 and *E. coli* C grew on Aga and GlcNAc (Figures 5B and 5D) and *E. coli* C grew on Gam (Figure 5C) but EDL933 did not grow on Gam (Figure 5C) because it is Aga^+^ Gam^-^ as explained earlier. Growth of EDL933 Δ*agaI* on Aga was not affected (Figure 5B). *E. coli* C Δ*agaI* also grew on Aga and Gam (Figures 5B and 5C) indicating that deletion of the intact *agaI* gene in *E. coli* C did not affect the utilization of these amino sugars just as Aga utilization was not affected in EDL933 Δ*agaI*. Growth on GlcNAc as carbon and nitrogen source was unaffected in Δ*agaI* mutants of EDL933 and *E. coli* C (Figure 5D) indicating that *agaI* is not involved in the utilization of GlcNAc. The utilization of Aga by EDL933 Δ*nagB* and that of Aga and Gam by *E. coli* C Δ*nagB* was unaffected (Figures 5B and 5C). To resolve, whether *agaI* and *nagB* substitute for each other as *agaA* and *nagA* do, Δ*agaI* Δ*nagB* mutants were examined for growth on Aga and Gam. As shown in Figure 5B, the utilization of Aga by EDL933 Δ*agaI* Δ*nagB* and that of Aga and Gam by *E. coli* C Δ*agaI* Δ*nagB* (Figures 5B and 5C) was not affected in these double knockout mutants thus providing convincing evidence that neither *agaI* nor *nagB* is required in the Aga/Gam pathway and particularly in the deamination and isomerization of Gam-6-P to tagatose-6-P and NH_3_. That Δ*nagB* and the Δ*agaI* Δ*nagB* mutants of EDL933 and *E. coli* C could not utilize GlcNAc (Figure 5D) was not unexpected as it is known that the loss of *nagB* affects GlcNAc utilization [2,4]. Identical results were obtained as in Figures 5B, 5C, and 5D, when these mutants were analyzed for growth on Aga, Gam, and GlcNAc plates without any added nitrogen source (data not shown). Complementation of Δ*nagB* and the Δ*agaI* Δ*nagB* mutants of *E. coli* C with pJFnagB restored growth of these mutants on GlcNAc containing NH_4_Cl thus showing that the inability of these mutants to grow on GlcNAc was solely due to the loss of *nagB* (data not shown)*.* In addition, we have also observed by phenotypic microarray [12,13] that utilization of GlcN, ManNAc, and N-acetylneuraminic acid was also affected in Δ*nagB* and Δ*agaI* Δ*nagB* mutants (data not shown) as catabolism of these amino sugars is known to lead to the formation of GlcN-6-P as a common intermediate [5]. Relative expression levels of *agaA, agaS, and nagA* were examined by qRT-PCR in these Δ*nagB* mutants following growth on glycerol and Aga. In glycerol grown Δ*nagB* mutants of EDL933 and *E. coli* C, *agaA, agaS,* and *nagA* were not induced. This is unlike Δ*nagA* mutants grown on glycerol where *nagB* was induced (Table 1). When grown on Aga, *agaA* and *agaS* were induced about 685-fold and 870-fold, respectively, in EDL933 Δ*nagB* and 150-fold and 90-fold, respectively, in *E. coli* C Δ*nagB*. These levels of induction are comparable to that in Aga grown Δ*nagA* mutants (Table 1). The *nagA* gene was not induced in Aga grown Δ*nagB* mutants. It was also examined if *agaI* on a multi-copy plasmid would complement Δ*nagB* and Δ*agaI* Δ*nagB* mutants for growth on GlcNAc. The plasmid, pJFagaI, did not complement these mutants of *E. coli* C for growth on GlcNAc even in the presence of 10, 50, and 100 μM IPTG (data not shown) indicating that *agaI* cannot substitute for the absence of *nagB*.

**Figure 6:**
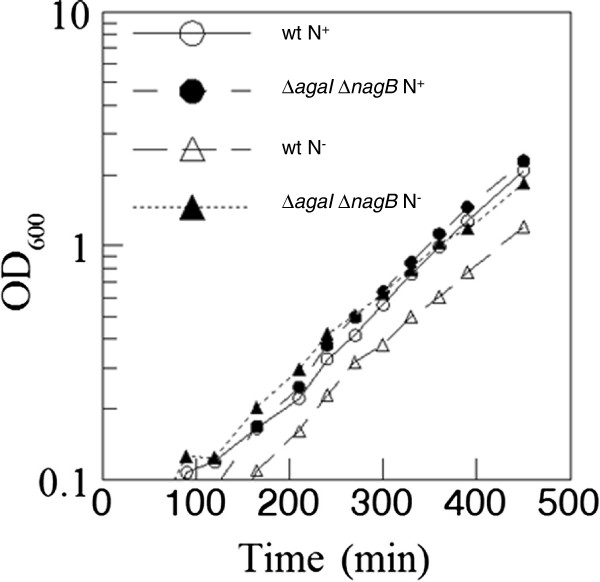
**Growth of EDL933 and EDL933 Δ*agaI *Δ*nagB *in Aga liquid medium with and without NH**_**4**_**Cl. **EDL933 (wt) and EDL933 Δ*agaI *Δ*nagB* were grown with shaking at 37°C in Aga MOPS medium with NH_4_Cl (N+) and without NH_4_Cl (N-). Growth (OD_600_) was monitored at indicated time intervals.

Growth rates of these mutants were measured in liquid MOPS minimal medium containing Aga with or without added NH_4_Cl in order to find if they would manifest growth rate differences compared to the wild type that otherwise cannot be detected by growth on plates. The doubling times of EDL933 and *E. coli* C in Aga MOPS medium with NH_4_Cl were about 80 and 115 min, respectively, and their doubling times without NH_4_Cl were about 90 and 135 min, respectively (data not shown for *E. coli* C) (Figure 6). The doubling times of the Δ*agaI*, Δ*nagB*, and Δ*agaI* Δ*nagB* mutants of EDL933 and *E. coli* C in Aga MOPS medium with and without NH_4_Cl were similar to that of their wild type parent strains (data not shown except for EDL933 and EDL933 Δ*agaI* Δ*nagB* in Figure 6). As seen from the slope of the plots there is no discernible difference in the doubling times of EDL933 Δ*agaI* Δ*nagB* on Aga with and without NH_4_Cl when compared with the doubling times of EDL933 in similar medium. The readings plotted in Figure 6 were from the exponential phase of growth of the cells and the growth curve for EDL933 without NH_4_Cl (N^-^) is slightly shifted to the right because of a longer lag phase but the slope is similar to that of EDL933 Δ*agaI* Δ*nagB* without NH_4_Cl. These growth experiments in liquid medium confirm the experiments done on plates (Figure 5).

The catalytic mechanism and the crystal structure of GlcN6-P deaminase/isomerase have been studied in detail [16-18] but to our knowledge there is only one report that showed that this enzyme was specific for only GlcN-6-P and Gam-6-P was unaffected [19]. Our studies with the ∆*nagB* mutant of EDL933 and particularly with ∆*agaI* ∆*nagB* mutants of EDL933 and *E. coli* C corroborate the lack of specificity of GlcNAc-6-P deaminase/isomerase for Gam-6-P. This is because had *nagB* been involved in the deamination and isomerization of Gam-6-P in strains where *agaI* is missing, then growth on Aga of EDL933 ∆*nagB* and EDL933 ∆*agaI* ∆*nagB* and growth on Aga and Gam of *E. coli* C ∆*agaI* ∆*nagB* would have been affected (Figure 5). In addition, as shown above, *agaI* cannot substitute for the absence of *nagB*, because pJFagaI could not complement Δ*nagB* and Δ*agaI* Δ*nagB* mutants of *E. coli* C*.* Together, these results show that *agaI* and *nagB* are not involved in Aga and Gam utilization. These results show that first three of the four proposals that we proposed above, do not hold true. Therefore, our fourth proposal that *agaI* and *nagB* are not essential for Aga and Gam utilization and that some other gene carries out the deamination/isomerization step holds true. So it poses the question which gene is involved in this step of the Aga/Gam pathway.

### The loss of *agaS* affects Aga and Gam utilization

**Figure 7:**
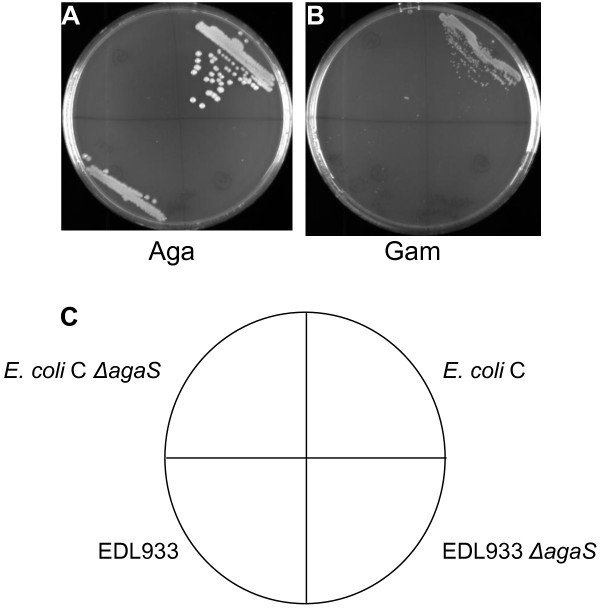
**Growth of EDL933, *E. coli *C, and Δ*agaS *mutants on Aga and Gam. **Wild type EDL933, *E. coli *C, and Δ*agaS* mutants derived from them were streaked out on MOPS minimal agar plates with Aga (**A**) and Gam (**B**) with NH_4_Cl as added nitrogen source. The Aga plate was incubated at 37°C for 48 h and the Gam plate was incubated at 30°C for 72 to 96 h. The description of the strains in the four sectors of the plates is indicated in the diagram below (**C**).

The *agaS* gene in the *aga/gam* cluster has not been assigned to any of the steps in the catabolism of Aga and Gam (Figure 1) [1,6]. Since *agaS* has homology to sugar isomerases [1] it was tested if deleting *agaS* would affect Aga and Gam utilization. EDL933 Δ*agaS* and *E. coli* C Δ*agaS*, did not grow on Aga plates but their parent strains grew (Figure 7A). On Gam plates, wild type *E. coli* C grew but *E. coli* C Δ*agaS* did not grow (Figure 7B). EDL933 and EDL933 Δ*agaS* were streaked on Gam plates but they were not expected to grow because EDL933 is Gam^-^ (Figure 7B). The results were identical when the Δ*agaS* mutants were examined for growth on Aga and Gam plates without any added nitrogen source (data not shown). These results show that the loss of *agaS* affects Aga and Gam utilization and therefore AgaS plays a role in the Aga/Gam pathway.

Relative expression levels of *nagA*, *nagB*, and *agaA* were examined by qRT-PCR in Δ*agaS* mutants grown on glycerol and GlcNAc. In glycerol grown Δ*agaS* mutants of EDL933 and *E. coli* C, *nagA*, *nagB*, and *agaA* were not induced. When grown on GlcNAc, *nagA* and *nagB* were induced about 10-fold and 23-fold, respectively, in EDL933 Δ*agaS* and 3-fold and 7-fold, respectively, in *E. coli* C Δ*nagB*. These expression levels of *nagA* and *nagB* in GlcNAc grown EDL933 Δ*agaS* are comparable to that in GlcNAc grown EDL933 Δ*agaA* (Table 1) but the levels of expression of these genes in GlcNAc grown *E. coli* C ∆agaS are lower than in GlcNAc grown *E. coli* C Δ*agaA* (Table 1). The *agaA* gene was not induced in GlcNAc grown Δ*agaS* mutants.

### Complementation studies with Δ*agaS* mutants

**Figure 8:**
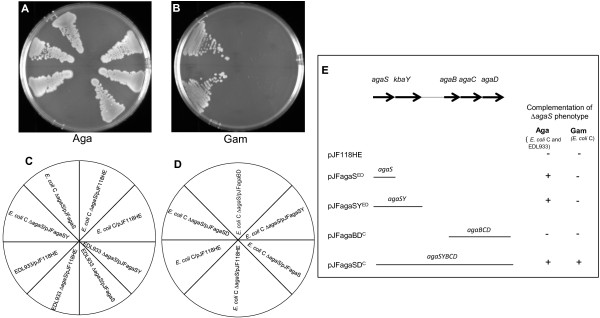
**Complementation of ∆*agaS *mutants of EDL933 and *E. coli *C on Aga and Gam plates. **EDL933 and *E. coli *C and the ∆*agaS *mutants derived from them harboring the indicated plasmids were streaked out on Aga MOPS minimal agar plate with NH_4_Cl (**A**) and containing ampicillin and incubated at 30°C for 72 h. For Gam complementation, *E. coli* C and *E. coli *C ∆*agaS *harboring the indicated plasmids were streaked out on Gam MOPS minimal agar plate with NH_4_Cl (**B**) and containing ampicillin and incubated at 30°C for 96 h. The strains with various plasmids in the different sectors of the plates in A and B are shown below in **C** and and **D**, respectively. The panel on the right (**E**) describes the various plasmids used for complementation of ∆*agaS* mutants and summarizes the results from the plates (**A** and **B**). The complementation results of EDL933 ∆*agaS*/pJFagaBD^C ^are not shown in plates **A **and **B**.

Complementation experiments were carried out to confirm that the deletion of *agaS* caused the Aga^-^ phenotype in EDL933 Δ*agaS* and the Aga^-^ Gam^-^ phenotype in *E. coli* C Δ*agaS* and not because this deletion was exerting a polar effect on downstream genes, namely, *kbaY*, *agaB*, *agaC*, *agaD*, and *agaI* (Figures 1 and 8E). Among these genes, *kbaY* is involved in the last step of the Aga and Gam pathway, while *agaBCD*, are involved in Gam uptake and *agaI* is not needed for the utilization of Aga and Gam as we have shown above. Thus, if the Aga^-^ phenotype in the Δ*agaS* mutants is due to a polar effect on a downstream gene it would be *kbaY.* As expected, the EDL933/pJF118HE and *E. coli* C/pJF118HE grew on Aga whereas the ∆*agaS* mutants with pJF118HE did not grow (Figure 8A). Importantly, *E. coli* C and EDL933 ∆*agaS* mutants with either pJFagaS^ED^ or pJFagaSY^ED^ grew on Aga (Figures 8A and 8E). Complementation of the Aga^-^ phenotype by pJFagaS^ED^ showed that deletion of *agaS* caused the Aga^-^ phenotype and not because the deletion of *agaS* had a polar effect on *kbaY* expression. Although both pJFagaS^ED^ and pJFagaSY^ED^ complemented the Aga^-^ phenotype they failed to complement the Gam^-^ phenotype in *E. coli* C ∆*agaS* (Figures 8B and 8E). It is likely that the deletion in *agaS* was causing a polar effect on *agaBCD*. This was tested by using pJFagaBD^C^ to complement the Gam^-^ phenotype. *E. coli* C ∆*agaS*/pJFagaBD^C^ did not grow on Gam plates (Figures 8B and 8E). The plasmid, pJFagaBD^C^, is functional because we have shown that EDL933 which is Gam^-^ manifests a Gam^+^ phenotype when it harbors this plasmid (unpublished data). Since neither pJFagaSY^ED^ nor pJFagaBD^C^ could complement the Gam^-^ phenotype, the most likely explanation is that the deletion of *agaS* not only affects the Aga/Gam pathway but also exerts polarity on the expression of *agaB*, *agaC*, and *agaD*. If this is the case, then the plasmid, pJFagaSD^C^, should complement the Gam^-^ phenotype and it does because *E. coli* C ∆*agaS*/ pJFagaSD^C^ grew on Gam plates (Figures 8B and 8E). Identical results were obtained when complementation was done on Aga and Gam plates without any added nitrogen (data not shown). These experiments raise the question why the partial deletion of *agaS* in ∆*agaS* mutants does not exert polarity on *kbaY* but is polar on further downstream *agaBCD* genes. The most likely explanation is that the strength of the polarity is a function of distance from the mutation [20,21]. These complementation experiments were done at 30°C because it was observed that at lower temperatures complementation of ∆*agaS* mutants with these plasmids was better. In addition, complementation by these plasmids was not observed when IPTG was added at a concentration as low as 10 μM (data not shown) suggesting that over-expression of the AgaS protein, unlike over-expression of AgaA and NagA, is detrimental to the cell. These experiments clearly demonstrate that the *agaS* gene is involved in Aga and Gam utilization.

### The *agaS* gene codes for Gam-6-P deaminase/isomerase

Since *agaI* is not involved in the Aga/Gam pathway, the only step in the Aga/Gam pathway that does not have a gene assigned to it is the deamination and isomerization of Gam-6-P to tagatose-6-P. On the other hand, the *agaS* gene is the only gene that has not been linked to any step in the Aga/Gam pathway [1,6]. It has been inferred that since the promoter specific for *agaS* is repressed by AgaR and *agaS* is inducible by Aga and Gam, AgaS must be involved in the catabolism of Aga and Gam [11]. Our results with the ∆*agaS* mutants confirm this (Figure 7).

The *agaS* gene is homologous to the C-terminal domain of GlcN-6-P synthase (GlmS) that has the ketose-aldose isomerase activity but does not have the N-terminal domain of GlmS that binds to glutamine [1]. The C-terminal domain of GlmS is found in a wide range of proteins that are involved in phosphosugar isomerization and therefore this has been named as the sugar isomerase (SIS) domain [22]. This SIS domain that is in AgaS has been shown to be present in prokaryotic, archaebacterial, and eukaryotic proteins [22]. Interestingly, a novel archaeal GlcN-6-P-deaminase which has been demonstrated to have deaminase activity is related to the isomerase domain of GlmS and has the SIS domain [23]. Proteins with SIS domains have been classified in the Cluster of Orthologous Group of proteins as COG222. It was proposed by Tanaka and co-workers that although AgaI has sequence homology to *nagB* encoded GlcNAc-6-P deaminase/isomerase and has been predicted to be the Gam-6-P deaminase/isomerase, AgaS which belongs to COG222 could be an additional Gam-6-P deaminase [23]. Based on these reports and our findings that neither *agaI* nor *nagB* has a role in Aga and Gam utilization, we propose that *agaS* codes for Gam-6-P deaminase/isomerase. In light of this proposal that *agaS* codes for Gam-6-P deaminase/isomerase, we tested if pJFnagB would complement *E. coli* C ∆*agaS* mutant for growth on Aga and similarly if pJFagaS would complement *E. coli* C ∆*nagB* mutant for growth on GlcNAc. In both cases, no complementation was observed even with 10, 50, and 100 μM IPTG (data not shown). Therefore, these results show that *agaS* and *nagB* function only in their respective pathway. Whether *agaI* serves as an additional deaminase/isomerase remains uncertain because over-expression of *agaI* from pJFagaI in *E. coli* C ∆*agaS* was unable to complement the Aga^-^ phenotype (data not shown).

## Conclusions

The Aga/Gam pathway has not been extensively studied as evidenced by the few publications that exist in the literature [1,6,9-11,24]. In this study we show that *agaI* is not needed for growth on Aga and Gam and *nagB* does not substitute for the absence of *agaI* as we had originally proposed [12]. Instead, we propose that the product of the *agaS* gene carries out this step. During the preparation of this manuscript, Leyn et al. published a paper that also showed that *agaI* is not essential for Aga utilization but *agaS* is essential [24]. Also, in a three-step enzyme coupled assay they showed that AgaS has deaminase activity and in a two-step assay they detected AgaA deacetylase activity [24]. In their experiments they observed complementation of the ∆*agaS* mutant with the *agaSY* and not with *agaS* alone as we have observed. This difference is most likely because they used *agaS* deletion mutants with a spectinomycin cassette that could cause a polar effect on *kbaY*. Furthermore, they carried out complementation in liquid medium whereas we did on agar plates at 30°C which could cause this difference. Additionally, we show that *agaA* is not essential for growth on Aga because *nagA* can substitute for *agaA* and that *agaA* and *nagA* can substitute for each other but, on the other hand, *agaS* and *agaI* cannot complement a ∆*nagB* mutant and neither can *nagB* complement a ∆*agaS* mutant. Interestingly, AgaA has only 10 fold lower activity with GlcNAc-6-P than with Aga-6-P whereas, AgaS has 27-fold lower activity with GlcN-6-P than with Gam-6-P [24] indicating that *agaA* could substitute for *nagA* but *agaS* is unlikely to substitute for *nagB* as we have shown. Therefore, our genetic data complements and supports the biochemical data on AgaA and AgaS. The Aga/Gam pathway as revealed from these studies is depicted in Figure 1 which shows that *agaS* and not *agaI* codes for Gam-6-P deaminase/isomerase. The interplay of AgaA and NagA but not that of AgaS and NagB between the Aga/Gam and GlcNAc pathways as revealed from this study is also indicated in Figure 1. What role, if any, *agaI* plays in the Aga/Gam pathway remains to be investigated.

## Methods

### Bacterial strains

*E. coli* O157:H7 strain EDL933 (FDA strain # EC1275) was from our collection of strains at the Food and Drug Administration. This strain is henceforth referred to as EDL933. *E. coli* strain C, strain # CGSC 3121, and all strains and plasmids for gene knockout experiments by the method of Datsenko and Wanner [25] were obtained from the Coli Genetic Stock Center at Yale University, New Haven, CT.

### Bacterial media and growth conditions

To test growth on minimal medium agar plates, wild type and the knockout mutant strains were grown overnight with shaking in Luria Broth (LB) at 37°C. The cultures were then diluted 10^3^ fold in 0.9% NaCl and streaked on MOPS modified buffer (Teknova, Hollister, CA) agar plates supplemented with 1.32 mM K_2_HPO_4_ and 0.001% yeast extract containing 20 mM of glucose, Aga, or GlcNAc. To test growth on glucose, Aga, and GlcNAc in nitrogen free medium everything was the same as described above except that MOPS modified buffer minus NH_4_Cl (Teknova) was used. To test growth on Gam plates with and without NH_4_Cl everything was the same as described above except that the concentrations of Gam and K_2_HPO_4_ were reduced by half to 10 mM and 0.0625 mM, respectively. In complementation experiments on plates, 100 μg/ml of ampicillin was added to the plates. Except where indicated, plates were incubated at 37°C for 48 h. For measurement of growth rate on Aga, wild type and knockout strains were grown overnight in MOPS liquid minimal medium with and without NH_4_Cl containing 20 mM Aga. The overnight cultures were diluted 100 fold into fresh medium and growth was monitored by measuring optical density at 600 nm (OD_600_) at indicated time intervals.

### Construction of knockout mutants

The *agaA*, *nagA, agaS, agaI*, and *nagB* chromosomal genes in EDL933 and *E. coli* C were disrupted following a standard method [25]. The *agaR* gene was deleted in *E. coli* C. The primers used for constructing knockout mutants are shown in Table 3. The knockout mutants constructed with the kanamycin cassette inserted and those with the kanamycin cassette eliminated were verified by PCR using appropriate primers flanking the target regions (Table 3). The mutants with the kanamycin cassette eliminated were further verified by DNA sequencing (Macrogen, Rockville, MD) using primers shown in Table 3. All knockout mutants used in this study were cured of their kanamycin cassettes except for the *agaR* knockout strains of *E. coli* C from which the kanamycin cassette was not removed. The whole *agaI* gene in *E. coli* C and similarly the whole *agaI* gene encompassing both the open reading frames (ORFs) in EDL933 were deleted creating *E. coli* C Δ*agaI* and EDL933 Δ*agaI*. The whole *nagB* gene was also deleted in both strains creating *E. coli* C Δ*nagB* and EDL933 Δ*nagB*. The double knockout mutants, EDL933 ∆*agaI* ∆*nagB* and *E. coli* C ∆*agaI* ∆*nagB* were constructed from their respective ∆*agaI* parents. The *agaA* gene coding for a 377 amino acid long Aga-6-P deacetylase in EDL933 was deleted from the 74th to the 209th codon. The identical region of *agaA* in *E. coli* C was deleted. The *nagA* gene coding for a 382 amino acid long GlcNAc-6-P deacetylase was deleted from 47th to the 334th codon in both *E. coli* C and EDL933. The double knockout mutants, EDL933 ∆*agaA* ∆*nagA* and *E. coli* C ∆*agaA* ∆*nagA* were constructed from their respective ∆*agaA* parents. The *agaS* gene coding for a 384 amino acid long AgaS protein in EDL933 was deleted from the 67th to the 314th codon and the identical region in the *agaS* gene of *E. coli* C was deleted. The *agaR* gene in *E. coli* C codes for a 227 amino acid long repressor protein and it was deleted from the 106th to the 183rd codon in *E. coli* C and *E. coli* C ∆*nagA*.

**Table 3:** List of primers used for constructing and verifying gene knockouts and gene cloning

Name^a^	Strain^b^	Sequence (5' to 3')
Primers for gene knockouts		
5agaA	Both	GGCGTTGATGTAATGGATGACGCGCCGGATGTACTCGACAATGGTGTAGGCTGGAGCTGCTTC
3agaA	Both	CTGCCGCATCAACAGACAGCGTACTGCCCGCCAG CCACCATTATTCCGGGGATCCGTCGACC
5nagA	Both	TAGCGGAACTGCCGCCAGAGATCGAACAACGTTCACTGAAAATGGTGTAGGCTGGAGCTGCTTC
3nagA	Both	AGGATGATATGTGGACCGGCAGCGACGATGTCGCTGCTTTATTATTCCGGGGATCCGTCGACC
5nagB	Both	AATCCGCCAACGGCTTACATTTTACTTATTGAGGTGAATAATGGTGTAGGCTGGAGCTGCTTC
3nagB	Both	AAATATTGCCCTGAGCAAGGAGCCAGGGCAGGGATAACAAATTATTCCGGGGATCCGTCGACC
5agaI	Both	TGTGCTCTCTATTGTTTGTTTCCGCATTCGGCATTTTGTAAATGGTGTAGGCTGGAGCTGCTTC
3agaI	EDL933	ATAAGTTAATTTAAACATTTTGAGCAATTTTTCATCTGGATTATTCCGGGGATCCGTCGACC
3agaI	*E. coli C*	GGCGACCCGCGGTTTTTAACATCTCATGTTGCTGTGTTCTATTATTCCGGGGATCCGTCGACC
5agaS	EDL933	TGCGGATCATCCTGACCGGAGCCGGAACCTCGGCATTTATATGGTGTAGGCTGGAGCTGCTTC
5agaS	*E. coli C*	CTGCGGATCATCCTGACCGGAGCCGGAACGTCGGCATTTATATGGTGTAGGCTGGAGCTGCTTC
3agaS	Both	AGGATGATATGTGGACCGGCAGCGACGATGTCGCTGCTTTATTATTCCGGGGATCCGTCGACC
5agaR	*E. coli C*	ACGCAGCGTTGCGAAAGCTGCCGTTGAGTTGATTCAGCCAATGGTGTAGGCTGGAGCTGCTTC
3agaR	*E. coli C*	CTGACGCCGCGCTCCAGATCGATCGCATCTACACCAAGAAATTATTCCGGGGATCCGTCGACC
Primers for PCR and sequencing for verification of gene knockouts		
FagaA	Both	ATGACACACGTTCTGCGCGCCAG
RagaA	Both	TCAAAACGAAGCTAATTGACCCTG
FnagA	Both	ATGTGGACCATCAGCTGTCTGC
RnagA	Both	TTCTTTGATCAGCCCGCGTTCGA
FnagB	EDL933	TATCGCAAATTAAACGAGTGTCT
RnagB	Both	GTTCAGTGAACGTTGTTCGATCTCT
FnagB	*E. coli C*	TATCGCAAATTAAACGCGTGTCT
RagaI	Both	TGACATTCGTTTGCCATCGACAGTAC
FagaI	EDL933	GACTTTGCTGCGCCAGGGGGCGAGT
RagaI	*E. coli C*	TGAGCAAATTTTTCATCTGGTTAGG
FagaS	Both	CATCCAGCAATCCTTTTGCTTC
RagaS	EDL933	TAGATCTCTTCCAGCGCGATATGTT
RagaS	*E. coli C*	TAGATCTCTTCCAGCGCGATGTGTT
FagaR	*E. coli C*	ATGAGTAATACCGACGCTTCAGGT
RagaR	*E. coli C*	ACCAGAATCACTTCAACCCCAGCC
Primers for cloning genes		
5nagAHindIII	Both	GCATAAGCTTACATTTTACTTATTGAGGTGAATAATGTATGCATTAACCCAGGGCCGGATC
3nagASmaI	Both	GCATCCCGGGTTATTGAGTTACGACCTCGTTACCGTTAA
5agaAHindIII	EDL933	GCATAAGCTTCAGTAATCTGAACTGGAGAGGAAAATGTCCGGTCGAGGAAGGGATATGACA
5agaAHindIII	*E. coli C*	GCATAAGCTTCAGTAATCTGAACTGGAGAGGAAAATGTCCGGTCGAGGAAGGAATATGACA
3agaAPstI	Both	GCATCTGCAGTCAAAACGAAGCTAATTGACCCTGAATCC
5agaIHindIII	*E. coli C*	GCATAAGCTTGTTCATCAGACTAAGGATTGAGTTATGGAACGAGGCACTGCGTCTGGTGG
3agaISmaI	*E. coli C*	GCATCCCGGGTTAAGGTGTTAATTAAACAAATAAAGTTC
5nagBHindIII	*E. coli C*	GCATAAGCTTACATTTTACTTATTGAGGTGAATA
3nagBSmaI	*E. coli C*	GCATCCCGGGTTACAGACCTTTGATATTTTCTGC
5agaSHindIII	EDL933	GCATAAGCTTGTTCATCAGACTAAGGATTGAGTT
3agaSPst1	EDL933	GCATCTGCAGTTATGCCTGCCACGGATGAATGATTACGC
3agaYPst1	EDL933	GCATCTGCAGTTATGCTGAAATTCGAATTCGCTG
5agaSDHindIII	*E. coli C*	TAGCATAAGCTTATGCCAGAAAATTACACCCCT
3agaSDEcoR1	*E. coli C*	TAGCATGAATTCTTACAAAATGCCGAATGCGGA
5agaBDHindIII	*E. coli C*	GCATAAGCTTGTTCATCAGACTAAGGATTGAGTTATGACCAGTCCAAATATTCTCTTAAC
3agaBDSmaI	*E. coli C*	GCATCCCGGGTTACAAAATGCCGAATGCGGAACAAACAA

^a ^The primer names indicate the genes that are targeted for construction and verification of knockout mutants and for cloning. The number, 5, and the letter, F, preceding the name of the gene indicate forward primers and the number, 3, and letter, R, preceding the name of the gene indicates reverse primers. Restriction enzymes used for cloning a gene are stated in the primer name following the name of the gene.

^b^ The strain name indicates the primer used for that particular strain and when the same primer is used for both strains it is indicated as both.

### Cloning experiments

All genes cloned in this study were amplified by PCR from EDL933 or *E. coli* C using appropriate primers as indicated in Table 3. The PCR fragments and the plasmid, pJF118HE [26], were digested with indicated restriction enzymes (Table 3) and cloned following standard protocols. In this study, the following genes were cloned into pJF118HE for complementation experiments: *agaA and nagA* were cloned from both EDL933 and *E. coli* C forming pJFagaA^ED^, pJFagaA^C^, pJFnagA^ED^, and pJFnagA^C^ (the superscripts, ED and C, indicate the strains EDL933 and *E. coli* C, respectively, from where the genes were cloned). The *agaI* and *nagB* genes were cloned from *E. coli* C resulting in pJFagaI and pJFnagB, respectively; *agaS* gene and the *agaSY* genes were cloned from EDL933 leading to pJFagaS^ED^ and pJFagaSY^ED^, respectively; and *agaB*C*D* and *agaSYBCD* genes were cloned from *E. coli* C resulting in pJFagaBD^C^ and pJFagaSD^C^, respectively. For complementation experiments, the parent vector, pJF118HE, and the recombinant plasmids were transformed into the indicated recipient strain by electroporation.

### RNA isolation and qRT-PCR

Wild type and mutant strains of EDL933 and *E. coli* C were grown overnight with shaking at 37°C in 30ml MOPS liquid minimal medium containing 20 mM of glycerol, Aga, or GlcNAc. The overnight cultures were diluted 100-fold into fresh medium and grown with shaking. When the cultures reached an OD_600_ between 0.6 and 0.7, 820 μl of cultures were withdrawn and mixed with 180 μl of chilled acidic phenol which were then centrifuged for 10 min at 4°C. The supernatants were discarded and the cell pellets were frozen immediately in a dry ice bath and stored at -70°C. RNA was isolated using RNeasy Mini Kit (Qiagen, Gaithersburg, MD) following the manufacturer’s instructions including the on-column DNA digestion step using DNase I. The integrity of the RNA was checked by running 1 μl of RNA using the Agilent RNA 6000 Nano Kit in an Agilent Bioanalyzer (Agilent Technologies, Santa Clara, CA). The RNA concentrations were measured using a NanoDrop spectrophotometer. Real-time RT-PCR was conducted using the iQ5 Optical System (Bio-Rad Laboratories, Hercules, CA). Each 20 μl reaction consisted of 50 ng RNA, 10 μl of 2x SYBR Green RT-PCR reaction mix, 1 μl of the iScript reverse transcriptase for one step RT-PCR, and 10 μl of 0.5 μM primer pairs. The first step in the real-time PCR program was a10 minute incubation at 50°C, followed by a 5 minute reverse transcriptase inactivation step at 95°C. This was followed by 40 cycles of 10 seconds of denaturation at 95°C and 30 seconds of annealing and elongation at the optimal annealing temperature for each specific primer pair (Table 4), during which fluorescence was measured. Next a melt curve analysis was included by increasing the temperature from 55 to 95°C in steps of 0.5°C for 10 seconds, when fluorescence was measured to allow the verification of the presence of one gene-specific peak. The cycle threshold (Ct value) was determined by the iQ5 Optical System Software from Bio-Rad Laboratories. All samples were run in duplicate and the average relative expression of each gene was normalized with the internal control gene, glyceraldehyde 3-P dehydrogenase (*gapA*) and the relative fold change was calculated using 2^-∆∆Ct^ method [27].

**Table 4:** List of primers used for qRT-PCR

**Name**^**a**^	**Strain**^**b**^	**Sequence**
5RTagaA	EDL933	CCGTTTCTCAGCACACCTTA
3RTagaA	EDL933	CCCAGCATCACTCGTACATT
5RTnagA	EDL933	TTACCTTTGCCACCCATCTG
3RTnagA	EDL933	GCAGGCCATCAGCGATAATA
5RTnagB	EDL933	ATCTGTTTATGGGCGGTGTAG
3RTnagB	EDL933	GAGTGTCATGAGTCAGGGTTT
5RTagaA	*E. coli* C	ACTTCACGCCGCAGAATAA
3RTagaA	*E. coli* C	GCTGAGAAACGGCAATCAAC
5RTagaR	*E. coli* C	ACGGTATGAACGTGGCTAATG
3RTagaR	*E. coli* C	CAGCCTGATCGCCGTAAA
5RTagaS	Both	ATCCGCTGCTGTTGATCTC
3RTagaS	Both	GGTGATAGCATTCCGGTACAA
5RTnagA	*E. coli* C	CCGTGGCTGAATCTGGTAAA
3RTnagA	*E. coli* C	ATGACGTCGGCGTTCTTAC
5RTnagB	*E. coli* C	ATCTGTTTATGGGCGGTGTAG
3RTnagB	*E. coli* C	GAGTGTCATGAGTCAGGGTTT
5RTgapA	Both	CGACCTGTTAGACGCTGATTAC
3RTgapA	Both	CGATCAGATGACCGTCTTTCAC

^a ^The primer names indicate the genes that are targeted for quantification of transcript. The number, 5 preceding the name of the gene indicate forward primers and the number, 3 preceding the name of the gene indicates reverse primers.

^b ^The strain name indicates the sequence used to design the primer was from that strain and when the same primer is used for both strains it is indicated as both.

## Authors’ contributions

ZH carried out the construction of knockout mutants, did cloning and other experiments, participated in the writing, and critically read the manuscript. IRP planned and conducted the quantitative real time RT-PCR experiments, analyzed the real time RT-PCR data, participated in the writing, and critically read the manuscript. AM conceived of the study, planned and did experiments, and wrote the manuscript. All authors read and approved the manuscript.
