# Correction: Genetic analysis of the roles of *agaA*, *agaI*, and *agaS* genes in the N-acetyl-D-galactosamine and D-galactosamine catabolic pathways in *Escherichia coli* strains O157:H7 and C

**DOI:** 10.1186/1471-2180-14-127

**Published:** 2014-05-30

**Authors:** Zonglin Hu, Isha R Patel, Amit Mukherjee

**Affiliations:** 1 Division of Molecular Biology, Office of Applied Research and Safety Assessment, Center for Food Safety and Applied Nutrition, U.S. Food and Drug Administration, Laurel, MD 20708, USA

## 

After the publication of this work [1], it was brought to our attention that in Figure 1B it is erroneously shown that Gam is transported by EII^Aga^ and Aga is transported by EII^Gam^. The correct depiction should be that Gam is transported by EII^Gam^ and Aga is transported by EII^Aga^ and the corrected figure is now shown in Figure 1.

**Figure 1:**
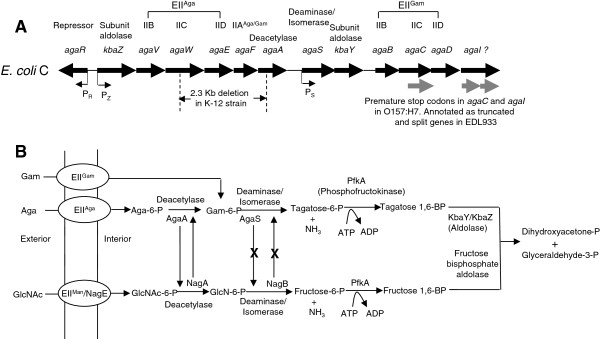
**The *aga***/***gam *regulon and the Aga**, **Gam**, **and GlcNAc pathways in *E. coli***. (**A**) The genetic map (not drawn according to scale) shows the 13 genes and the protein products that they code for in the 12.3 Kb *aga*/*gam* cluster in *E. coli* C. *The agaI* gene was predicted to code for Gam-6-P deaminase/isomerase but this study and that of Leyn et al. [24] show that *agaS* codes for this deaminase. The question mark next to *agaI* indicates that the function of this gene is now uncertain. P_R.,_ P_Z,_ and P_S_ are the promoters and the arrows indicate the direction of transcription. The 2.3 Kb deletion in the K-12 strain is shown and the truncated *agaC* gene and the split *agaI* gene as annotated in strain EDL933 are shown in gray arrows. (**B**) The Aga/Gam and the GlcNAc pathways are depicted in this figure. The only change from what was known before about the Aga/Gam pathway [1], [6] is that AgaS carries out the deamination step and not AgaI as was known before. The GlcNAc pathway is shown to indicate the interplay between AgaA and NagA but not between AgaS and NagB as shown from this study. The upward vertical arrow from NagA indicates that it can substitute for AgaA and the downward vertical arrow indicates that AgaA can substitute for NagA when it is over-expressed. The upward vertical arrow from NagB with an X in the middle and a similar downward arrow from AgaS indicate that AgaS and NagB do not substitute for each other.

We regret any confusion caused by this error. We wish to thank Dr. Jacqueline Plumbridge for bringing this error to our attention.
